# Pre-clerkship EPA assessments: a thematic analysis of rater cognition

**DOI:** 10.1186/s12909-022-03402-x

**Published:** 2022-05-06

**Authors:** Eric G. Meyer, Emily Harvey, Steven J. Durning, Sebastian Uijtdehaage

**Affiliations:** 1 Department of Psychiatry, Uniformed Services University of the Health Sciences, 4301 Jones Bridge Rd, Bethesda, MD 20814 USA; 2 The Henry M. Jackson Foundation for the Advancement of Military Medicine, Bethesda, MD USA; 3 Department of Medicine, Center for Health Professions Education, Bethesda, MD USA

**Keywords:** Competency based assessment, Entrustable professional activities, Rater cognition

## Abstract

**Background:**

Entrustable Professional Activities (EPAs) assessments measure learners’ competence with an entrustment or supervisory scale. Designed for workplace-based assessment EPA assessments have also been proposed for undergraduate medical education (UME), where assessments frequently occur outside the workplace and may be less intuitive, raising validity concerns. This study explored how assessors make entrustment determinations in UME, with additional specific comparison based on familiarity with prior performance in the context of longitudinal student-assessor relationships.

**Methods:**

A qualitative approach using think-alouds was employed. Assessors assessed two students (familiar and unfamiliar) completing a history and physical examination using a supervisory scale and then thought-aloud after each assessment. We conducted a thematic analysis of assessors’ response processes and compared them based on their familiarity with a student.

**Results:**

Four themes and fifteen subthemes were identified. The most prevalent theme related to “student performance.” The other three themes included “frame of reference,” “assessor uncertainty,” and “the patient.” “Previous student performance” and “affective reactions” were subthemes more likely to inform scoring when faculty were familiar with a student, while unfamiliar faculty were more likely to reference “self” and “lack confidence in their ability to assess.”

**Conclusions:**

Student performance appears to be assessors’ main consideration for all students, providing some validity evidence for the response process in EPA assessments. Several problematic themes could be addressed with faculty development while others appear to be inherent to entrustment and may be more challenging to mitigate. Differences based on assessor familiarity with student merits further research on how trust develops over time.

**Supplementary Information:**

The online version contains supplementary material available at 10.1186/s12909-022-03402-x.

## Introduction

Competency-based, time-variable education is a hotly debated topic in medical education [1, 2]. In response to criticisms that current models for assessment in competency-based medical education (CBME) are too reductionist and onerous, [3, 4] ten Cate and Scheele introduced a synthetic assessment framework based on entrustable professional activities (EPAs) that are assessed with trust [5]. EPAs are “professional activities that together constitute the mass of critical elements that operationally define a profession” [5]. An EPA assessment is operationalized by how much supervision an assessor believes the learner requires to safely execute the activity. Studies suggest that a single EPA assessment, combined with narrative feedback, can serve a formative purpose, helping a learner understand their current performance and driving improvement [6]. Furthermore, studies have found that a robust collection of EPA assessments for multiple tasks, in conjunction with other assessment data, can be employed in a program of assessment [7] and inform high-stake advancement determinations [8].

EPA assessments are typically done in the clinical workplace and resemble the decisions supervisors make frequently regarding their trainees [9]. They are intended to align with how clinicians think, fit in to the daily work flow [10], and thus seem to ask “the right questions, in the right way, about the right things” [11]. EPA assessments are supposed to reflect a trainee’s ability to do a task, but it has been theorized that in workplace-based assessment (WBA) they are also influenced by factors beyond the control of the trainee: the characteristics of the assessor, the context in which the trainee was observed, the task itself, and the relationship between trainee and assessor [12, 13]. It has been further theorized that such trust itself develops overtime – from presumptive and initial trust to grounded trust [12, 13]. These characteristics not only re-enforce the complex nature of rater cognition [14], but also hint at the intricacies of trust, which has historically been conceptualized as a multidimensional concept [15, 16]—an intuition, and perhaps even a gut feeling [17].

While initially intended for residency training, there has also been interest in using EPAs in early medical training [18]. To that end, EPA tasks for entering clerkship [19] and an entrustment scale specific to undergraduate medical education (UME) have been developed [20], both of which were met with skepticism [21]. Moreover, given that opportunities for workplace-based assessments in the pre-clerkship phase of medical training are often simulated, infrequent, or absent altogether - it is unclear how assessors arrive at an entrustment rating in those circumstances. All of this raises questions about the validity evidence of the decisions that are based, in part, on EPA assessments.

To assess the validity of an assessment, it has been proposed that a series of four step-wise inferences must be supported by evidence: *scoring* (translating an observation into a score or scores); *generalization* (interpreting the score or scores as a reflection of test performance); *extrapolation* (interpreting the score or scores as a reflection of real-world performance), and *implications* (making an advancement decision based on the score or scores) [22, 23]. The first inference, scoring, is further elucidated in Messick’s work [24]: the scoring inference is highly dependent on how well the assessors response process, which is highly linked to rater cognition [14], aligns with the task being assessed. It has been recently argued that entrustment has good construct alignment with the task actually being assessed as preliminary evidence in support of the scoring evidence of EPA assessments [25]. This is a critical assertion, as a compromised scoring inference would undermine the generalization (e.g., a combination of entrustment scores effectively represent performance in the test setting such as OSCEs), extrapolation (e.g., OSCE performance predicts performance in the clerkships), and implication (e.g., the decisions based on the assessment have the desired effect such as, medical students’ clinical skills are sufficiently developed to participate in patient care during clerkships) in EPA assessments [22, 23].

While previous work has sought evidence to elucidate the scoring inference of specific EPA tasks in UME [26], validity evidence pertaining to the scoring inference, and more specifically the response process, for EPA assessments remains sparce [18]. Evidence for the scoring inference must support the claim that high entrustment scores reflect good ability to perform an EPA, and low scores reflect low ability. Furthermore, one could argue that when two students execute an EPA equally well, they should receive the same entrustment score if they are held to the same standards. We don’t know the extent to which assessors adjust their scores based on performance they witnessed previously. This could complicate the interpretation of entrustment scores if they no longer reflect *just* the observed performance. Furthermore, if entrustment scores are based, in part, on thought processes that are incompatible with fair and accurate assessment, subsequent inferences, and indeed, the validity evidence of the entire assessment process may become questionable.

In this qualitative study, we explored evidence for the scoring inference by examining the response process [24] and, more specifically, the rater cognition [14] of faculty as they observe pre-clerkship students interviewing standardized patients (SP). Additionally, given that grounded trust is thought to develop over time [12], a secondary aim of this study was to compare assessors’ thought processes based on their familiarity with a student’s prior performance..

## Methods

We employed a qualitative approach using a think-aloud protocol [27] to better understand what influences faculty entrustment decisions outside of a workplace-based setting [28]. We then conducted a thematic analysis [29] of the transcripts generated from these think-alouds. After gaining ethics approval from the USUHS IRB (Protocol # DBS.2019.046), assessors were asked to think aloud while making entrustment determinations based on their observations of students completing a task.

### Context

USUHS students practice the task of completing a history and physical examination prior to starting the clerkship year in a course called Introduction to Clinical Skills (ICS). The students meet with their faculty five times over 18-months. Groups of six students work with a longitudinal preceptor who observes them working with standardized patients in a simulated environment – like the workplace-based environments they will encounter during clerkship [30, 31]. These preceptors provide students with formative and summative feedback. ICS culminates in a final, summative Objective Structured Clinical Exam (OSCE), consisting of four clinical stations where, in addition to other skills, students demonstrate their ability to complete a history and physical examination. These OSCE encounters are each 20 min long and are video recorded for quality assurance purposes. Performance was scored by trained standardized patients [32] and was the sum of checklist history items, physical exam maneuvers, and a communication score [33].

### Participants

Sample size was initially set at nine faculty to ensure a variety of clinical specialties (e.g., internal medicine, pediatrics), expertise in teaching (e.g., junior and senior faculty, residency directors and instructors), and genders. This sample size would later be reassessed based on our ability to reach thematic saturation or sufficiency [34]*.* We recruited nine longitudinal preceptors via email from a pool of 32 potential faculty who had each worked with six ICS students over the previous 18-months. For each participant, we selected one video depicting the performance of one of their own longitudinal students (a “familiar student”) and one video of a student whom they had never observed before (an “unfamiliar student”). Videos were selected based on checklist performance to ensure there was a wide range of performance across the sample and that performance was comparable between students familiar and unfamiliar to a participant. This resulted in nine participants (henceforth referred to as “assessors”) watching two videos from a sample of nine videos – one of a familiar student and one of an unfamiliar student. This difference was not highlighted to assessors to avoid keying their thought process.

### Assessor task

Raters were asked to watch a video of a pre-clerkship medical student completing a history and physical examination. The task of completing a history and physical examination was defined using to the Association of American Medical Colleges’ (AAMC) Core EPA 1: Completing a History & Physical Examination [35]. Core EPA 1 was chosen because it performed well on the EQual rubric [26, 36] and, as a task, is easily understood by assessors as a key skill all medical students must master before starting the clerkships. Assessment of the student was accomplished with the Chen supervisory scale, which is designed for student’s entering clerkship [20]. This nine-item scale measured entrustment using levels of supervision as a surrogate, ranging from “1a: Not allowed to observe” to “5: Allowed to supervise others” [20]. Confidence in these assessments was measured using a four-item scale: no confidence, low confidence, intermediate confidence, high confidence. Confidence was included as a means for assessing any change in the development of trust (from initial to grounded) [12, 13] that may not have resulted into a change in entrustment / supervisory levels.

One author (EH) began each session by obtaining informed consent. After consenting to participate, the assessors were provided with two handouts. The first contained the basic info on Core EPA 1 (H&P) from the AAMC EPA Guide [37] (Online Supplement 1). The second handout was a grid of the Chen supervisory scale [20] (y-axis) and the confidence scale (x-axis) (Online Supplement 2). The assessors were instructed to point at the box on the grid that corresponded with “the level of supervision they thought the student required along with their confidence in their decision.” To reduce bias, no guidance regarding prior knowledge of a student was provided. Similarly, language [20] guidance regarding determining current or future supervisory requirements was also left out to avoid keying assessor thinking. Assessors were asked to update their assessment as frequently as they changed either their entrustment determinations or confidence – or if new data emerged that confirmed their previous assessment.

### Data collection

Following the think-aloud protocol [27], we asked assessors to make repeated interim entrustment assessments as the student’s OSCE video was playing. We paused the video each time the assessor made an assessment to reduce the cognitive load of simultaneously watching the student, assessing the student, and thinking-aloud [38]. This protocol allowed the assessor time to share their thinking that informed their assessment before the video resumed. If more than 4 min elapsed before or between assessments, we manually paused the video to ask the assessor “think aloud” and make an assessment. Ensuring a think-aloud occurred at least every 4 min reduced pressure for the accessor to recall all their thinking at the end of the encounter. It also provided a better understanding of the interactive and iterative phases of rater cognition where the rater is repeatedly observing, processing, and integrating [14]. This method was piloted with a separate assessor to refine the process.

Rather than asking the assessors to describe their thought process in terms of pre-existing theories related to entrustment [12, 13], which may have restricted our ability to detect novel response processes [39] or rater cognitions [14], we provided no framework for the assessors and simply asked them to “think-aloud.” All assessors practiced thinking-aloud with a separate five-minute sample video prior to watching their two assigned videos. Each time the assessor made an assessment the video was paused, the author recorded the time on the video, the assessor’s assessment, and said, “Please think-aloud.” These think-alouds were audio recorded. When the assessor was done with each think-aloud the video resumed. At the end of the video the assessor was asked to make a “final, overall determination” and to again think-aloud. Audio files were transcribed with NVivo© 12 (QSR International, Mar 2020).

### Data analysis

We conducted a six-step thematic analysis [29] of all transcripts set using an inductive approach. We first acknowledged our own backgrounds as physician educators (EM, SD), educators, education experts (SU, SD), and as a researcher (EH). We also explored our perceptions of trust and entrustment as important contextual lenses. We explored how these backgrounds might inform our interpretation of the data – and how the combination of our different backgrounds might provide balance. Our positionality was reviewed periodically to ensure our reflexivity was not causing undue influence on our analysis. After familiarizing ourselves with all 18 transcripts, four random transcripts were iteratively coded by three authors (EM, EH, SU), comparing results until there was consensus on a codebook. This codebook was then presented to the remaining author (SD) for refinement. Informed by this codebook, two authors (EM & EH) coded five additional transcripts. If any new codes emerged all nine sample transcripts would be re-coded looking for this new code. Once consensus was achieved, these initial nine transcripts were reviewed by the other authors (SU and SD) for agreement. When agreement was achieved, the remaining nine transcripts were coded (EM & EH). Again, coding was discussed between the two coders until there was consensus and an additional coder (SU or SD) was available if needed. If new codes emerged in this second set of transcripts all transcripts would be re-coded.

When all coding was complete the final codebook was semantically analyzed by the entire author team to identify and define potential themes and subthemes. These definitions were then reviewed against the entire data set to ensure they were representative and to facilitate further refinement. Lastly, key quotes that represented each theme and subtheme were selected and compiled.

As a further description of the themes [40], the prevalence was calculated using the frequency that transcripts included a theme or subtheme with the assistance of NVivo© 12 (QSR International, Mar 2020). Overall percentages were calculated for themes and percentages within a theme were calculated for subthemes. In addition to overall prevalence, we compared the prevalence of themes and subthemes in transcripts when assessors observed a familiar student versus an unfamiliar student.

## Results

Nine longitudinal preceptors from ICS course were recruited: five were Internists; three were Family Medicine physicians, and one was a Pediatrician. On average, they had been teaching medical students for 14 years (range: 5–38 years). Three had been clerkship directors and two had been residency program directors. Five were women, and four were men. Each reviewed a video of one of their recent longitudinal mentees (familiar student), and one video of a student they had not worked with previously (unfamiliar student). Of the nine student videos selected, five of the students were men and four were women. The average checklist performance score (as assessed by SPs) was 63.5% (range: 46–80%). Seven of the assessors completed their observations and think-alouds in person with an author (EH) during a single session that typically lasted about an hour. Due to the 2019 COVID pandemic, the final two participants completed the process via GoogleMeet (Google, Mountain View, CA).

When an assessor was familiar with the student, they provided on average 8.6 entrustment determinations and think-alouds per 20-min video (range: 5–22) compared to an average of 9.2 determinations (range: 5–22) when they had not observed the student previously. As listed in Table 1, the most common *initial* entrustment rating (made within the first 4 min) when assessors were familiar with a student was: “2b: *With supervisor in room ready to step in as needed*” which was slightly lower than the most common initial entrustment rating made by assessors unfamiliar with a student: “3a: *With supervisor immediately available, ALL findings double checked.*” The most common final, overall entrustment rating remained the same as the initial rating for both groups, but the range of entrustment ratings narrowed when assessors were familiar with students (2b - 3b) compared to when they were unfamiliar with students (2a - 5). The most common confidence ratings increased to “high confidence” in both groups, although when assessors were familiar with student’s prior performance they had a wider range of confidence [2–4] than when they were unfamiliar student’s prior performance (3–4).

**Table 1:** Entrustment and confidence ratings

Assessors’ previous familiarity with student	Initial Assessment		Final Assessment	
	Entrustment Mode (range)	Confidence Mode (range)	Entrustment Mode (range)	Confidence Mode (range)
Familiar	2b (2b - 5)	3 (2–4)	2b (2b - 3b)	4 (2–4)
Unfamiliar	3a (2a - 5)	3 (2–4)	3a (2a - 5)	4 (3–4)

Key: Entrustment - 2a: As coactivity with supervisor, 2b: With supervisor in room ready to step in as needed, 3a: With supervisor immediately available, ALL findings double checked, 3b: With supervisor immediately available, KEY findings checked, 5. Allowed to supervise others in practice of EPA. Confidence – 2: Low, 3: Intermediate, 4: High

The average length of each transcript was approximately 1500 words and each think-aloud was 150–300 words in length. Three rounds of iterative coding of four transcripts were required to develop an initial codebook. No new codes emerged after these initial four transcripts, confirming our sample size of 18 transcripts was likely to achieve thematic saturation (34). Fifteen subthemes emerged 764 times during the coding process, which were further organized into four themes. Definitions for each theme/subtheme are available in Table 2.

**Table 2:** Themes & subthemes found in think-alouds

**STUDENT PERFORMANCE (504, 65%)**	
*** Observable or inferred student activities - often described as skills.***	
1) Student behavior (362, 72%)	
These statements describe what the student is doing. They typically include an evaluation of the performance as good or bad - but are occasionally neutral observations.	
2) Inferred clinical reasoning (100, 20%)	
How or what the assessor explicitly perceives the student to be thinking. This is frequently based on a conclusion about what the student is doing / has done.	
3) Patient rapport (42, 8%)	
This represents the assessor’s understanding of the relationship between the patient and the student. It often appears that out of concern for a compromised relationship or rapport that more supervision would be needed, and that a good relationship can compensate for poor performance in other domains.	
**FRAME OF REFERENCE (123, 16%)**	
*** How the assessor understands the task at hand to include personal context or differences in understanding related to the purposes of the assessment.***	
4) Future training needs (40, 33%)	
The assessor considers how much supervision the student will require in the future rather than how much supervision they currently require.	
5) Assessor preference/self (20, 16%)	
These assessments/impressions are based on self - the assessor’s personal opinion or their preferred/historical way of doing the task.	
6) Affective response (20, 16%)	
When the assessor references their emotional state/reaction as part of their decision making.	
7) Phase of training (17, 14%)	
The assessor is using a phase of training to determine what level of entrustment/supervision is appropriate rather than the student’s performance.	
8) Previous exposure to the *same* student (13, 11%)	
The assessor incorporates knowledge and impressions regarding performance based on previous experiences with that same student.	
9) Comparison with *another* student’s performance (7, 06%)	
The assessor is utilizing a normative style of assessment, comparing the student to another student’s performance.	
10) The curriculum (6, 5%)	
The assessor believes that the student is doing something incorrectly and ascribes this to the curriculum (i.e., the assessors believe that the students was taught incorrectly). In light of this, they consider the behavior acceptable.	
**ASSESSOR UNCERTAINTY (88, 12%)**	
*** When the assessor questions their ability observe the student adequately.***	
11) Assessor confidence in their ability to assess (40, 45%)	
The assessor questions their own observations, but not due to an actual compromise of information or insufficient number of assessments. This often seems to be an attempt to couch a judgment. Note that assessor commented at least every 4 min, so it is unlikely that they actually had recall issues.	
12) Compromised Information (27, 31%)	
The assessor was not able to adequately observe a student due to camera position or time running out.	
13) Insufficient number of assessments (21, 24%)	
The assessor mentions that the number of times they have worked with the student is compromising their assessment, namely, that they haven’t worked with the student enough to make an entrustment assessment.	
**THE PATIENT (49, 6%)**	
*** Details specific to the patient, like acuity and risk associated with care.***	
14) Patient characteristics (27, 45%)	
The assessor considers patients characteristics, complexity, symptomatology in entrustment decisions without consideration of the student’s ability or patient safety.	
15) Patient safety (22, 55%)	
The assessor considers patient safety in their entrustment rating of the student. This has *less* to do with how the student is performing, and *more* to do with the potential risk to the patient associated with their signs/symptoms. For example, if an assessor reports concern because a student missed a critical question or physical exam maneuver, this would be coded as student behavior. This subtheme represents a fear that the patient may suffer (despite being simulated) regardless of the student’s performance	

N refers to how many times the subtheme/theme appeared in the transcripts. Theme percentage refers to total frequency. Subtheme percentages refers to the frequency within a theme

### Student performance

Observable or inferred student activities - often described as skills. This theme included “student behaviors,” “inferred clinical reasoning,” and “patient rapport.” and represented two-thirds of transcribed content (66%). Student behavior, as a subtheme, indicated that assessors was commenting on something they observed the student doing. This was frequently described in neutral terms, (e.g., “the student is asking about past medical history”) but was also occasionally described as correct (e.g., “they asked the key history questions”) or incorrect (e.g., “they failed to listen to the heart”). Clinical reasoning was typically inferred from what the student was doing:

*[These questions] assure me that [the student is] thinking about potentially red flag issues that might have brought the patient in.* ^(Assessor A, Unfamiliar student)^

or when the assessor questions the students’ clinical reasoning:

*I'm not sure where [this is] all going.* ^(Assessor B, Unfamiliar Student)^

Student rapport related to how the assessor understood the patient to be relating to the student. This code had two variants: patient response,

*The patient seems comfortable with him as well*. ^(Assessor A, Unfamiliar Student)^

and student effort:

*The student [is] using active listening and summarizing to build rapport*. ^(Assessor A, Unfamiliar Student)^

### Frame of reference

How the assessor understands the task at hand to include personal context or differences in understanding related to the purposes of the assessment. The next most common theme included seven different subthemes: “future training requirements,” “Assessor preference/self,” “affective reactions” to the student’s performance, the “student’s phase of training,” “previous exposure to student performance,” “comparison with other students,” and “the curriculum.” Future training requirements revealed assessors were considering what supervision was needed in the future versus what supervision was required during the current encounter. For example, most of the time the assessors commented on what supervision the student currently required:

*I would have wanted to be in the room and at least initially to be able to jump in*. ^(Assessor G, Familiar student).^

Occasionally, however, they discussed levels of supervision in the future tense – indicating they were considering what level of supervision the student would require in the future:

*She'd have to work with a supervisor before she could conduct this stuff in an independent fashion.* ^(Assessor E, Unfamiliar student)^

Assessor preference/self was used when the assessor referred to themselves as frame of reference:

*I would have put this patient in the chair and done the interview in the chair to make it a little more relaxing*. ^(Assessor H, Unfamiliar student)^

Assessors’ affective response appeared to manifest as disappointment, pain, feeling good/better, discomfort and surprise:

*In the past I’ve seen her do this incredibly well so I’m a little surprised that she didn’t do as well on this [case]*. ^(Assessor H, Familiar student)^

Phase of training represented when entrustment decisions were informed by where the student was in training rather than just their performance:

*But there's part of me that also is inhibited by the fact that he's a second-year medical student.* ^(Assessor B, Familiar Student)^

An exemplar of how familiarity with a student’s prior performance can influence entrustment was:

*[My rating is] based partly on my past-experience with her and what I've observed this time*. ^(Assessor H, Familiar student)^

Conversely, a lack of familiarity also impacted entrustment:

*Right now, I'm putting low confidence just because I don't know the student and I'm realizing that does make an impact.* ^(Assessor D, Unfamiliar student)^

Assessors occasionally compared the student in the second video to the student from the first video. Of note, the order of the videos was randomized (familiar/unfamiliar) and this subtheme did not appear more often for either type of relationship.

*So, she's doing a better job than the last student characterizing the complaint - she seems to be more methodical*. ^(Assessor G, Unfamiliar student)^

The curriculum was evoked as a frame of reference for what the assessors expected. Occasionally, it was referenced as an “excuse” for the student not performing as expected. For example, this assessor is referencing a perceived inadequacy in the curriculum, despite assessing a student they longitudinally instructed:

*Based on what I know about the curriculum here [...] I wonder if she may not have the ability to form a broad differential for that [chief complaint].* ^(Assessor A, Familiar Student)^

### Assessor uncertainty

When the assessor questions that their ability to observe the student adequately. The third theme included “assessor confidence” in their ability to assess, “compromised information,” and concern regarding an “insufficient number of assessments.” Assessor confidence was commonly expressed near the end of the student’s performance and reflected their uncertainty about their own ability to assess aspects of a student’s performance without giving an explicit explanation:

*I still don't know what his level of knowledge is… and I don't know about his clinical decision making…so I think he's able to conduct the interview, but not necessarily*. ^(Assessor G-Familiar student)^

In contrast, compromised information had to do with an inability to observe what the student was doing, typically due to the camera angle of the video:

*I can’t tell, but it does not look to me like the bed is at a 45 degree angle… it looks much flatter because we’re looking down*. ^(Assessor H-Unfamiliar student)^

Insufficient number of assessments referred to an entrustment score being limited because of a single observation and the need for further exposure to the student:

*Given the opportunity to observe [the student] several more times, I might be willing to fairly quickly move him up in the in the supervisory level scheme. But just with one […] single observation, [I] wouldn't go any higher just yet*. ^(Assessor A-Unfamiliar Student)^

### The patient

Details specific to the patient, like acuity and risk associated with care*.* This least common theme related to “patient safety” and “patient characteristics.” These subthemes did not relate to the quality of the student’s performance. For example, if a student neglected to ask a critical question, failed to complete an important part of the physical exam, or neglected a diagnostic option that might threaten patient safety, these errors were considered student performance. Patient safety, as a subtheme, was noted regardless of student performance:

*The patient wasn't in extremis, so I don't think that the supervisor needed to be in the room.* ^(Assessor F-Familiar student)^

Similarly, “patient characteristics” had little to do with student performance, but instead typically highlighted an assessor’s desire to see “such a complicated patient” before they “left the clinic.”

Comparison of the subthemes we identified when assessors observed students with whom they were familiar with to those they were unfamiliar with revealed several key differences (Table 3). When assessors were unfamiliar with a student, “self” as a frame of reference subtheme was more prevalent (6/9 vs 3/9 videos) as was the lack of confidence in their ability to assess subtheme (7/9 vs 4/9). When assessors were familiar with a student, the subtheme related to referencing previous experiences with a student was more prevalent (6/9 vs 0/9) along with the affective response subtheme (7/9 vs 4/9). The concern regarding an insufficient number of assessments subtheme was comparable for both groups: assessors desired additional opportunities to assess familiar students in three out of nine videos compared to five out of nine videos of unfamiliar students.

**Table 3:** Frequency of themes & subthemes as organized by faculty familiarity with the student

	No Longitudinal Relationship (***N*** = 9)		Longitudinal Relationship (***N*** = 9)	
	Number of references (*N* = 407)	Number of transcripts the theme/subtheme appeared in (N = 9)	Number of references (*N* = 357)	Number of transcripts the theme/subtheme appeared in (*N* = 9)
**Student Performance**	**275 (68%)**	**9 (100%)**	**229 (64%)**	**9 (100%)**
Student behavior	198 (49%)	9 (100%)	164 (46%)	9 (100%)
Inferred clinical reasoning	57 (14%)	9 (100%)	43 (12%)	8 (89%)
Patient rapport	20 (4%)	5 (56%)	22 (6%)	6 (67%)
**Frame of Reference**	**56 (14%)**	**7 (78%)**	**67 (19%)**	**9 (100%)**
Future training needs	20 (5%)	4 (44%)	20 (6%)	6 (67%)
Assessor preference / “self”	16 (4%)	6 (67%)	4 (1%)	3 (33%)
Affective response	6 (1%)	4 (44%)	14 (4%)	7 (78%)
Phase of training	6 (1%)	5 (56%)	11 (3%)	7 (78%)
Previous exposure to the *same* student	0 (0%)	0 (0%)	13 (4%)	6 (67%)
Comparison with *another* student’s performance	5 (1%)	2 (22%)	2 (1%)	1 (11%)
The curriculum	3 (1%)	2 (22%)	3 (1%)	2 (22%)
**Assessor Uncertainty**	**55 (14%)**	**8 (89%)**	**33 (9%)**	**7 (78%)**
Assessor confidence in their ability to assess	27 (7%)	7 (78%)	13 (4%)	4 (44%)
Compromised information	16 (4%)	6 (67%)	11 (3%)	7 (78%)
Insufficient Number of Assessments	12 (3%)	5 (56%)	9 (3%)	3 (33%)
**The Patient**	**21 (5%)**	**8 (89%)**	**28 (8%)**	**8 (89%)**
Patient safety	8 (2%)	6 (67%)	14 (4%)	6 (67%)
Patient characteristics	13 (3%)	5 (56%)	14 (4%)	6 (67%)

## Discussion

We examined the thought processes of assessors who were tasked with making an entrustment determination while they observed a student completing a history and physical examination with a standardized patient and identified four themes and fifteen subthemes that represent the assessors’ response processes. Subthemes represented all three phases of the rating process: observing, processing, and integrating [14]. The predominant theme and the main source of information on which assessors based their entrustment scores was, appropriately, *student performance*. While it is reassuring that student performance played a large role in how assessors arrived at an entrustment score, our assessors also employed several other considerations that went beyond the observed behavior and were outside a student’s realm of control. When assessors’ considerations do not directly relate to a student’s observed performance, evidence that entrustment scores are fair and accurate may be compromised.

Several of the subthemes in this theme represent challenges associated with observation-based clinical assessment [41]. The use of “faculty preference/self”, “phase of training”, and “comparison to other students” are pitfalls that have been recognized by others [6] and could be addressed with faculty development. To prepare faculty for direct observation assessment, performance dimension training and frame of reference training has been recommended [6] as this promotes a shared mental model and consistent application of performance standards. This raises the question: “Is frame of reference training for something as complex and intuitive as ‘trust’ possible?” The alternative, requiring assessors to revert back to tracking specific student behaviors (e.g., washing their hands, use of open-ended questions, correct physical exam maneuvers) and to use behaviorally anchored rating scales for scoring student performance, puts us back to “square one,” as this was regarded as a cumbersome and reductionist approach to assessment [3, 4] and was one of the arguments [11] for introducing EPA assessments in the first place.

One potentially problematic subtheme influencing rater cognition that appeared to be unique to entrustment assessments was “future training needs.” Assessors appeared to have varying interpretations regarding what they were assessing – the student’s current supervisory requirements versus their future supervisory requirements. The Modified Chen Scale is intended to assess future supervision requirements, as indicated by the typical preamble to the scale: “If you were to supervise this student again in a similar situation, which of the following statements aligns with how you would assign the task?” [20]. Without this prompt, assessors appeared to predominantly describe current supervision. It has recently been theorized that current/retrospective entrustment determinations are more behaviorally based while future/prospective entrustment determinations are more holistic and more likely to include the risk associated with different levels of supervision [42]. The impact of having a retrospective vs prospective perspective when making entrustment determinations represents an area for further research in which a deliberate theoretical framework and the purported purpose of the assessment should inform the research question. Validity theory [22, 43] would likely prioritize that the assessment be informed by the student’s performance while criteria for a good assessment [44, 45] might emphasize other aspects, such has how effect the assessment catalyzes improvement.

Assessor familiarity with a student appeared to directly influence several aspects of rater cognition: “previous exposure to the same student,” “affective response,” and “accessor uncertainty.” It has previously been hypothesized that assessors need to consider previous observations of a student in order to develop grounded trust [12, 13] and that without a longitudinal relationship developing trust will be difficult [46]. It may be for these reasons that faculty were more likely to report being “uncertain” when rating students they were unfamiliar with. However, awareness of poor past performance has also been shown to negatively influence current assessments [47], which may explain the differences in entrustment assessments seen in this study (Table 1). Relatedly, “affective response” was typically disappointment that a student did not meet expectations. These differences impacted by student familiarity highlight an important consideration in the claim that entrustment scores accurately represent the competence demonstrated by a student in a patient encounter. If one believes that longitudinal preceptors are better positioned to do an ad-hoc EPA assessment because their trust, or lack thereof, is grounded in previous experiences with the student, one could argue that entrustment assessment should not be made by individuals who are unfamiliar with a student. However, if one believes that an ad-hoc assessment of a student should not be colored by a student’s past and should afford a fresh opportunity to excel, one could argue the opposite: EPA assessments ought to be done by a naïve assessor “without baggage.” The appropriateness of allowing previous knowledge of a student’s performance to influence an entrustment rating appears to be a matter of debate and warrants further research. Given the pros and cons associated with both types of assessment (longitudinal and naïve), it may be beneficial to employ a combination of the two.

As previously theorized [12, 13], patient-related factors influenced rater cognition when making entrustment assessments. In a GME context, training is embedded in real-life patient care and thus “patient safety” and “patient characteristics” must be considered when determining how much supervision a learner requires [48]. In UME, weighing these factors in non-workplace-based assessments is less intuitive because patient characteristics are deliberately selected and there is no risk of patient harm. Nonetheless, such situational factors are a critical part of understanding a student’s capabilities. Requiring assessors to describe the characteristics of the patient (such as diagnosis, acuity, and complexity) as well as the setting of the assessment, clarifies the context in which the assessment took place. Communicating the context and justification of the assessment also could help others, including students and competency committees that make advancement decisions, to appreciate the limitations of entrustment scores and the extent to which they can be generalized to other contexts.

This study has several limitations. First and foremost, it was done in a research setting and thus may not wholly represent the actual thinking that occurs as faculty are observing their students in real practice. Second, think-alouds have not been shown to be effective at detecting unconscious processing [27], so the themes may be missing important aspects of rater cognition. Third, these results may not generalize to work-based assessments in UME, when student interact with real patients. Furthermore, it is possible that are differences in thought processes when assessments are done through video compared to those done in person. Fourth, we may have missed biases or other factors that may have inappropriately influenced the performance ratings if our research participants chose not to verbalize them. Finally, to avoid influencing the assessors’ thought process, we did not instruct participants on whether previous exposure to a student should be considered and did not clarify if an entrustment determination was specific to current needs or future needs. This may have led to varying interpretations of the purpose the EPA assessments which could explain the wide range of entrustment scores. For example, some assessors thought pre-clerkship medical students were ready to supervise others completing a history and physical examination—a level of entrustment that seems far from what is appropriate in UME.

Work remains to be done to determine if EPA assessments outside of the workplace “ask the right questions, in the right way, about the right things” [11]. The most commonly employed themes we found in our study were related to student performance suggesting that scores represent student capability. This is an encouraging finding that supports the claim that EPA assessment scores obtained in a non-workplace-based setting (i.e., an OSCE) are aligned with the construct they purportedly measure: a students’ ability to execute an EPA. That said, several themes we identified in assessors’ response process suggest a misalignment between how some assessors arrive at an entrustment score and the purported domain of interest. Future research needs to clarify if these findings are evidence against the scoring inference and the extent to which they threaten the entire validity argument. While some of these concerns could be mitigated with faculty development, there are several that stem from the use of entrustment/supervision as an assessment framework, and from uncertainty of how trust is operationalized in the context of UME. Moreover, our field must decide whether it is appropriate to consider previous performance in EPA assessments.

## Supplementary Information

**Additional file 1.**

**Additional file 2.**

## Data Availability

All manuscripts and codes are available upon request. Please contact Dr. Meyer at eric.meyer@usuhs.edu.

## Abbreviations

EPA: Entrustable Professional Activity
UME: Undergraduate Medical Education
GME: Graduate Medical Education
WBA: Workplace Based Assessment
