# Supplementary material for: Pre-clerkship EPA assessments: a thematic analysis of rater cognition
Source: BMC Med Educ. 2022 May 6;22:347. doi: 10.1186/s12909-022-03402-x (PMC9077896; doi:10.1186/s12909-022-03402-x)
Supplement: Supplementary file 1 — Additional file 1. [file 12909_2022_3402_MOESM1_ESM.docx]

**Online Supplement 1:** Description of AAMC Core EPA 1 provided to study participants.

**Core EPA 1: Gather a history and perform a physical examination**

Day 1 residents should be able to perform an accurate complete or focused history and physical examination in a prioritized, organized manner without supervision and with respect for the patient. The history and physical examination should be tailored to the clinical situation and specific patient encounter. This data gathering and patient interaction activity serves as the basis for clinical work and as the building block for patient evaluation and management. Learners need to integrate the scientific foundations of medicine with clinical reasoning skills to guide their information gathering.

Functions

History

• Obtain a complete and accurate history in an organized fashion.

• Demonstrate patient-centered interview skills (attentive to patient verbal and nonverbal cues, patient/family culture, social determinants of health, need for interpretive or adaptive services; seeks conceptual context of illness; approaches the patient holistically and demonstrates active listening skills).

• Identify pertinent history elements in common presenting situations, symptoms, complaints, and disease states (acute and chronic).

• Obtain focused, pertinent histories in urgent, emergent, and consultative settings.

• Consider cultural and other factors that may influence the patient’s description of symptoms.

• Identify and use alternate sources of information to obtain history when needed, including but not limited to family members, primary care physicians, living facility, and pharmacy staff.

• Demonstrate clinical reasoning in gathering focused information relevant to a patient’s care.

• Demonstrate cultural awareness and humility (for example, by recognizing that one’s own cultural models may be different from others) and awareness of potential for bias (conscious and unconscious) in interactions with patients.

Physical Exam

• Perform a complete and accurate physical exam in logical and fluid sequence.

• Perform a clinically relevant, focused physical exam pertinent to the setting and purpose of the patient visit.

• Identify, describe, and document abnormal physical exam findings.

• Demonstrate patient-centered examination techniques that reflect respect for patient privacy, comfort, and safety (e.g., explaining physical exam maneuvers, telling the patient what one is doing at each step, keeping patients covered during the examination).

From AAMC CEPAERS’ Faculty & Learner Guide (Accessed Oct 2019)

https://store.aamc.org/downloadable/download/sample/sample_id/66/%20
