# Supplementary material for: Pre-clerkship EPA assessments: a thematic analysis of rater cognition
Source: BMC Med Educ. 2022 May 6;22:347. doi: 10.1186/s12909-022-03402-x (PMC9077896; doi:10.1186/s12909-022-03402-x)
Supplement: Supplementary file 2 — Additional file 2. [file 12909_2022_3402_MOESM2_ESM.docx]

**Online Supplement 2:** Table provided to faculty while overserving video performance.

|  | **Not**  **Confident** | **Low**  **Confidence** | **Intermediate Confidence** | **High**  **Confidence** |
| --- | --- | --- | --- | --- |
| 1a: Not allowed to observe EPA |  |  |  |  |
| 1b: Allowed to observe EPA |  |  |  |  |
| 2a: As coactivity with supervisor |  |  |  |  |
| 2b: With supervisor in room ready to step in as needed |  |  |  |  |
| 3a: With supervisor immediately available, ALL findings double checked |  |  |  |  |
| 3b: With supervisor immediately available, KEY findings checked |  |  |  |  |
| 3c: With supervisor distantly available (e.g., by phone), findings REVIEWED |  |  |  |  |
| 4. Allowed to practice EPA unsupervised |  |  |  |  |
| 5. Allowed to supervise others in practice of EPA |  |  |  |  |
